# Burkitt Lymphoma Presenting as Obstructive Jaundice: A Case Report and Literature Review

**DOI:** 10.7759/cureus.46215

**Published:** 2023-09-29

**Authors:** Kyle Madison, Zack Morgan, Wareef Kabbani

**Affiliations:** 1 Internal Medicine, Methodist Health System, Dallas, USA; 2 Surgical Pathology, Methodist Health System, Dallas, USA

**Keywords:** b cell lymphoma, 8q24 gene rearrangement, myc gene rearrangement, burkitt lymphoma, lymphoma, burkitt

## Abstract

Burkitt lymphoma (BL) is an aggressive, high-grade B-cell lymphoma common in children and young adults. Despite being frequently discovered in extranodal sites, BL rarely occurs in the pancreas. We present a case of a patient with BL presenting as obstructive jaundice.

## Introduction

Burkitt lymphoma (BL) is an aggressive type of non-Hodgkin lymphoma characterized by its rapid growth, propensity for extranodal involvement, and association with Epstein-Barr virus (EBV). BL typically presents with widespread lymphadenopathy, constitutional symptoms, and involvement of extranodal sites including the jaw, gastrointestinal tract, and bone marrow. However, in rare instances, BL can manifest with unusual clinical features, including obstructive jaundice, obscuring the diagnosis for physicians. Obstructive jaundice can result from various etiologies impeding the bile ducts. The occurrence of obstructive jaundice as the primary presenting symptom of Burkitt lymphoma is exceedingly rare, making it a diagnostic challenge for physicians. Atypical presentations, like obstructive jaundice, delay diagnosis and appropriate management, impacting patient outcomes. We present a case of a patient with BL presenting as obstructive jaundice and a literature review of non-Hodgkin lymphoma cases presenting similarly.

## Case presentation

**Figure 1:**
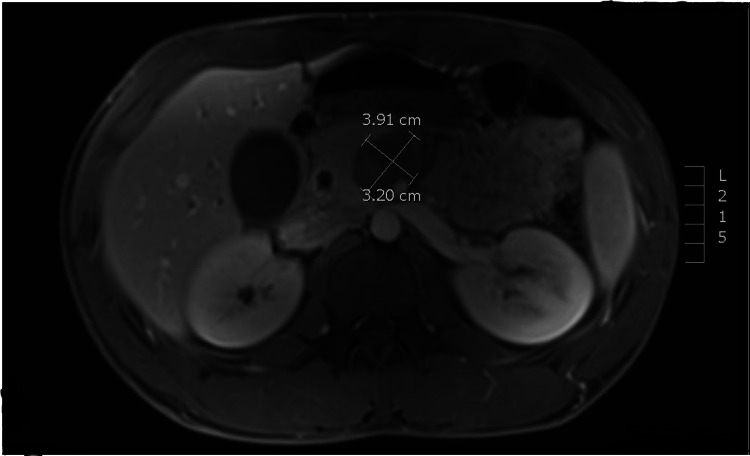
Magnetic resonance cholangiopancreatography showed a mild dilatation of the intrahepatic bile ducts, a lesion in the head of the pancreas, and a larger lesion in the uncinate process of the pancreas.

A 23-year-old male with no past medical history presented to the emergency department (ED) multiple times with left arm pain, suspected to be due to a muscular injury, and was treated with tramadol and meloxicam. On his first ED visit, a computed tomography (CT) scan of the abdomen and pelvis with contrast was obtained due to elevated transaminases and showed no acute intra-abdominal pathology. During subsequent ED evaluations, the elevated transaminases persisted. He again presented to the ED, this time with a scleral icterus. An abdominal ultrasound showed a small amount of sludge in the gallbladder with mild diffuse gallbladder wall thickening. He was admitted for further evaluation of his worsening transaminases and hyperbilirubinemia. Magnetic resonance cholangiopancreatography showed a mild dilatation of the intrahepatic bile ducts, which was thought to be secondary to a lesion in the head of the pancreas. A second, larger lesion was also seen in the uncinate process of the pancreas (Figure 1).

**Figure 2:**
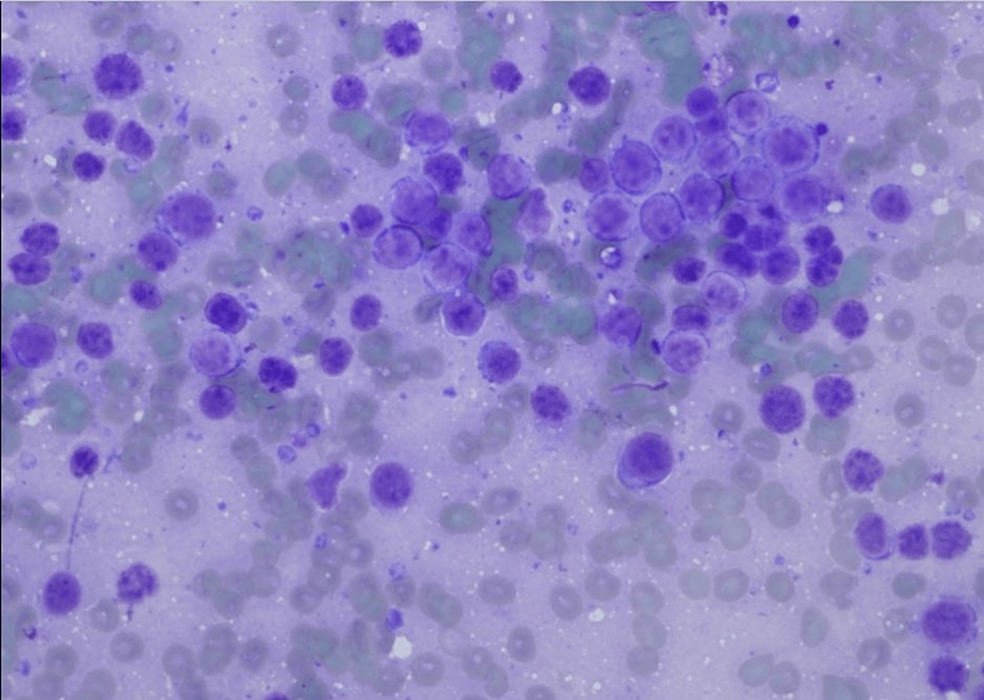
Direct smear from the EUS guided FNA, Diff Quik stain (X200). Note a population of immature lymphoid cells with intermediately sized nuclei. EUS: endoscopic ultrasound; FNA: fine-needle aspiration

**Figure 3:**
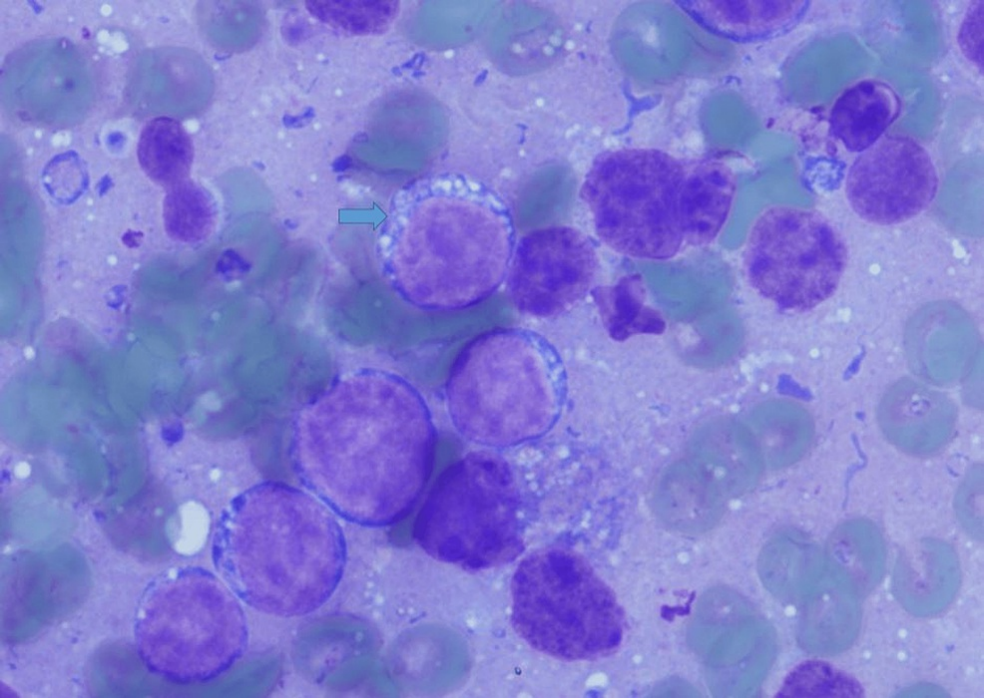
Direct smear from the EUS guided FNA, DiffQuik stain (X400). Note the immature appearance of the tumor cells with scant cytoplasm showing lipid vacuoles (arrow). EUS: endoscopic ultrasound; FNA: fine-needle aspiration

**Figure 4:**
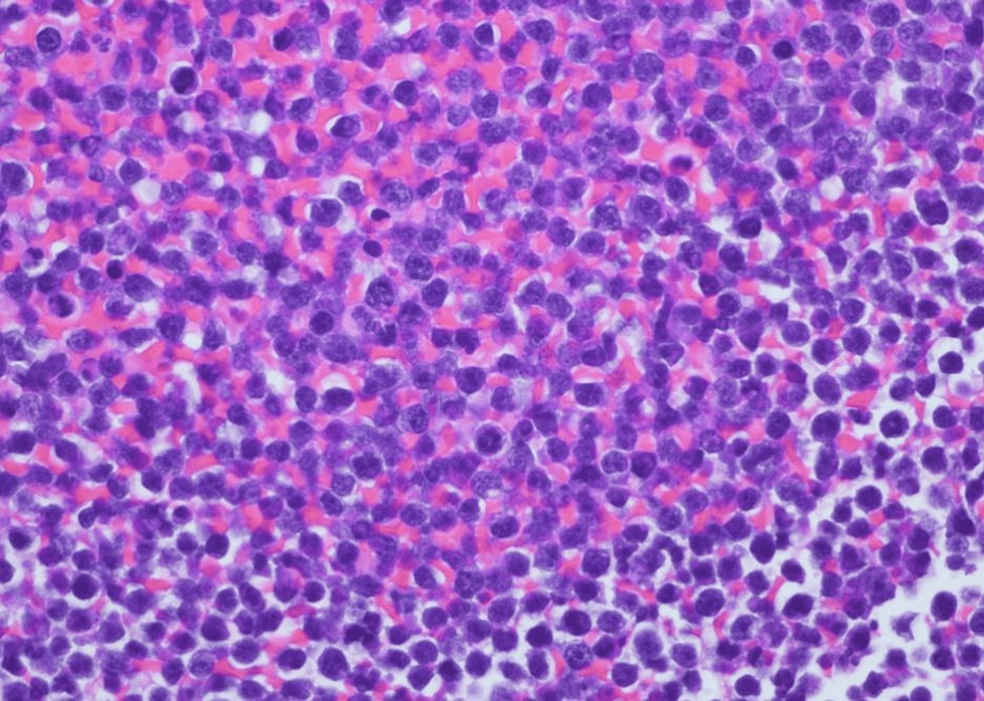
Cell block, H&E (x400). H&E: hematoxylin and eosin

Endoscopic retrograde cholangiopancreatography was subsequently performed, and two stents were placed, which resulted in an improvement in the jaundice. An endoscopic ultrasound-guided fine needle aspiration of the mass in the pancreatic uncinate process was performed. Examination of the direct smears and cell block preparations shows sheets of monotonous intermediate-sized cells with a starry sky appearance (Figures 2, 3, 4).

**Figure 5:**
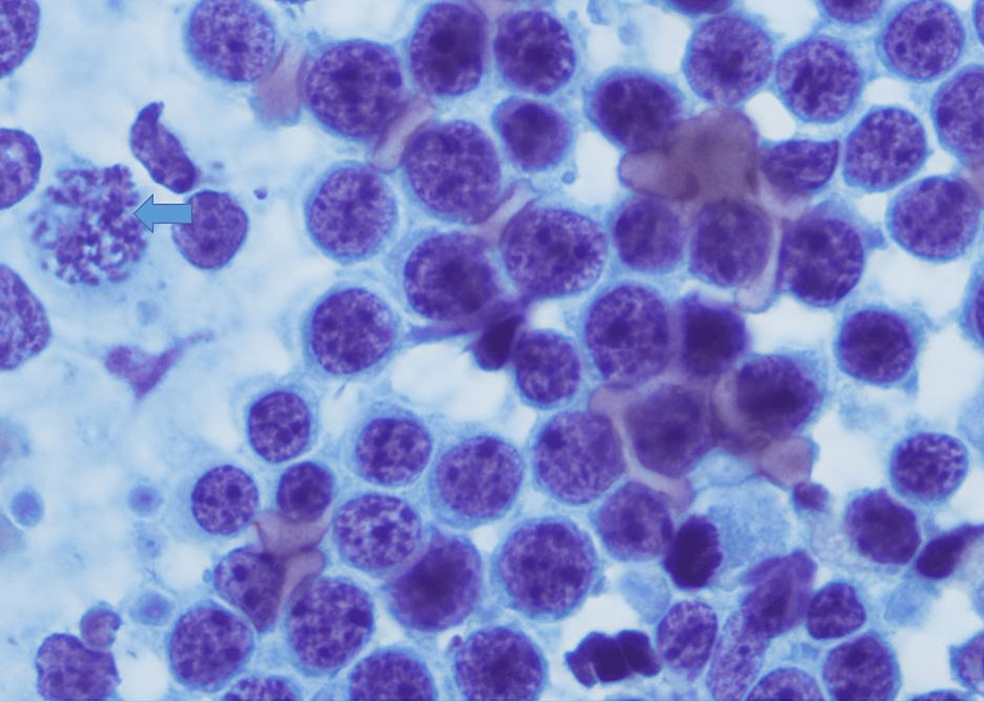
Direct smear from the EUS guided FNA, Papanicolau stain (X400). Notice the neoplastic cells with clumped chromatin and increased mitotic activity (arrow). EUS: endoscopic ultrasound; FNA: fine-needle aspiration

Numerous tingible body macrophages are noted and account for the starry sky morphology. The neoplastic cells demonstrate round nuclei with finely clumped chromatin and several paracentral nucleoli (Figure 5).

**Figure 6:**
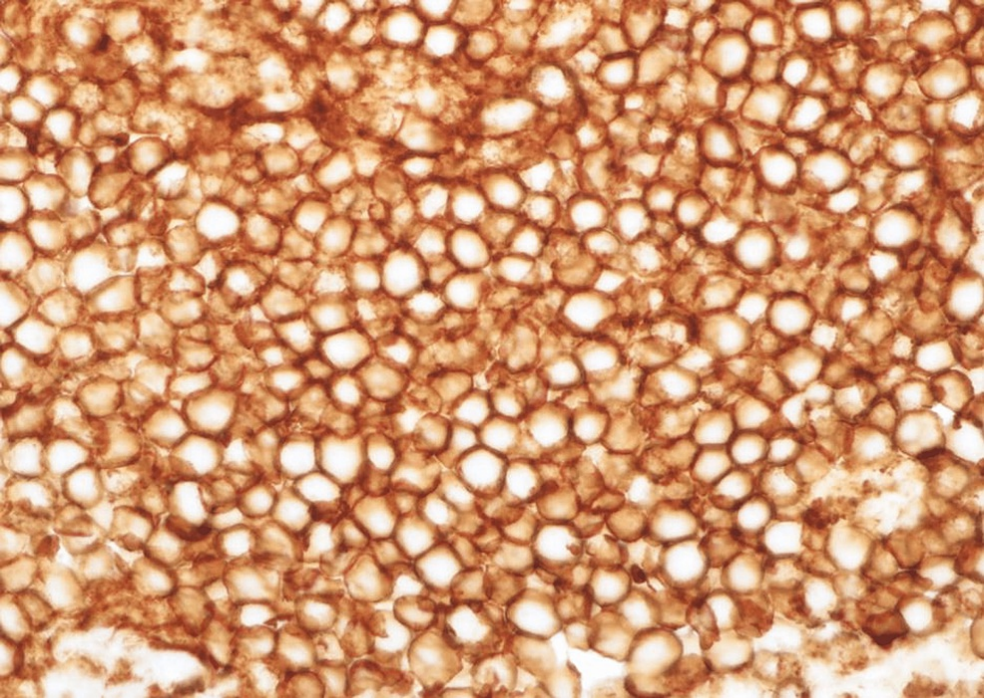
CD20 immunostain (X400).

**Figure 7:**
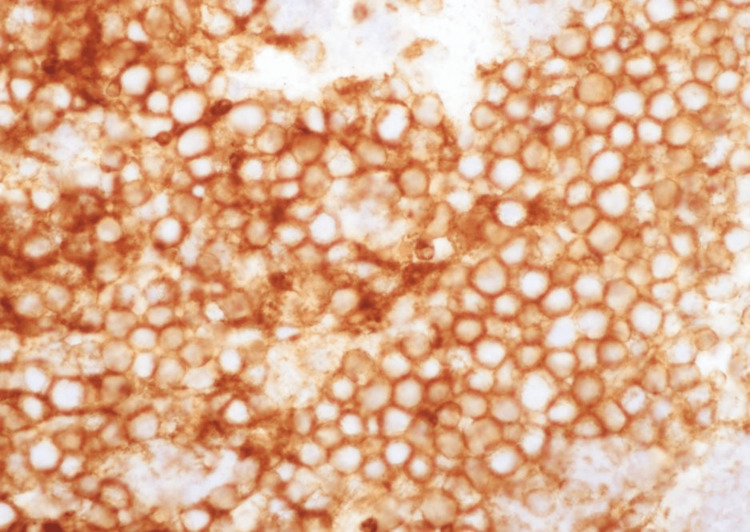
CD10 immunostain positive (X400).

**Figure 8:**
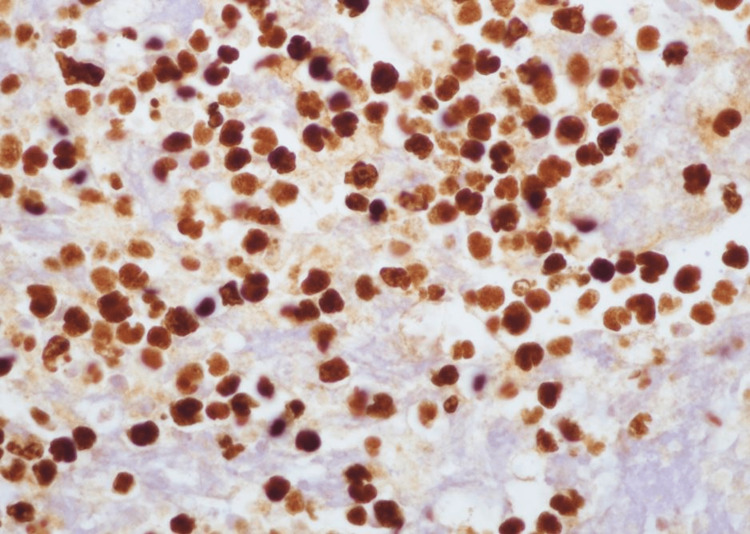
Epstein-Barr encoding region in situ hybridization positive in tumor cells (X400).

The mitotic rate is high with single-cell necrosis and increased apoptotic activity. Ancillary immunohistochemical staining was performed on the cell block, showing the neoplastic cells to express CD20, CD10, Bcl-6, and EBV (Figures 6, 7, 8).

**Figure 9:**
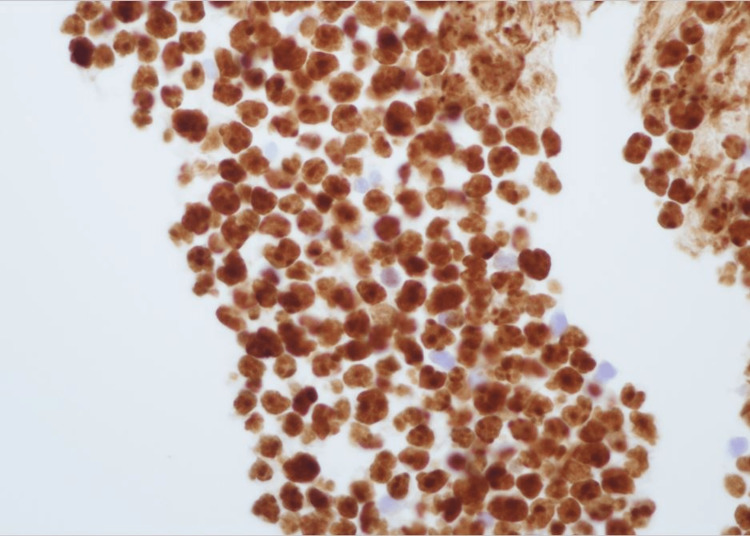
Ki67 proliferation index highlights more than 99% of tumor cells (x400).

The Ki-67 proliferation index was >99% (Figure 9).

Fluorescence in situ hybridization (FISH) analysis showed abnormal results with evidence of MYC (8q24) gene rearrangement and t(8;14)(q24;q32) in 87% and 88% of cells examined, respectively. FISH analysis was negative for BCL2 (18q21) and BCL6 (3q27) gene rearrangements, which helped exclude other high-grade B-cell lymphomas. There was evidence of right cervical lymph node involvement in addition to the two extranodal pancreatic masses consistent with stage IV BL. The patient was started on hyper-cyclophosphamide, vincristine, doxorubicin (Adriamycin), dexamethasone (CVAD), and rituximab, as well as intrathecal methotrexate and cytarabine. Three months after starting therapy, repeat positron emission tomography/computed tomography (PET/CT) showed resolution of lymph node disease and pancreatic masses.

## Discussion

BL is an aggressive, high-grade B-cell lymphoma most commonly found in children. It only accounts for <5% of lymphomas in adults [1]. Different clinical forms of BL are sporadic, endemic (associated with Epstein-Barr virus), or associated with immunodeficiency [2]. The sporadic form typically has an abdominal presentation, whereas the endemic form presents as a jaw or facial bone tumor. The pancreas is a rare extranodal presentation of BL, comprising less than 1% of extranodal lymphomas [3]. BL is associated with overactivation of the c-MYC proto-oncogene [4]. This can occur due to a t(8;14) translocation, which involves the Ig heavy chain locus on chromosome 14 and the c-MYC oncogene on chromosome 8.

Early tumor classification is essential for patients to receive prompt and appropriate treatment. For instance, surgical resection is the only potential curative option for early pancreatic adenocarcinoma, which typically presents in a 60- to 80-year-old patient population. Whereas primary pancreatic lymphoma (PPL) is usually diagnosed at a mean age of 53 years old [5], chemotherapy is the recommended first-line treatment [6]. Chemotherapy is the first line over surgical intervention in primary pancreatic BL since total pancreatomies do not improve overall survival [7]. As tumor classification determines intervention strategy, an intra-abdominal presentation of sporadic BL should be included in the differential for obstructive jaundice, especially in younger patients.

A literature review was conducted to identify similar cases of NHL presenting with obstructive jaundice. Six of the ten cases were biopsy-confirmed BL, with the remaining four cases identified as other B-cell lymphomas. Anatomically, five of the ten cases were peripancreatic lesions; two involved the common bile duct; one had a hepatic etiology of disease; and two had duodenal disease causing obstruction. The reviewed cases have been summarized in Table 1.

**Table 1:** Summary of non-Hodgkin lymphoma cases identified by literature review. BL: Burkitt lymphoma; NHL: non-Hodgkin lymphoma

Case Number	Patient Demographics	Clinical Summary	Reference article
1	Six-year-old male	The patient presented with jaundice and epigastric pain. He was found to have a pancreatic head mass. The biopsy was consistent with BL.	[8]
2	16-year-old female	The patient presented with biliary obstruction due to a duodenal neoplasm. Biopsy confirmed BL.	[9]
3	21-year-old female	The patient presented with obstructive jaundice, and was diagnosed with primary NHL of the common bile duct.	[10]
4-6	One adult and two children	Patients with peripancreatic NHL presented with obstructive jaundice.	[11]
7	14-year-old male	The patient presented with painless, pruritic jaundice and weight loss. The patient was diagnosed with ampullary BL.	[12]
8	12-year-old male	The patient presented with scleral icterus, weight loss, and painless abdominal distention. The patient was diagnosed with BL presenting as hepatic disease.	[13]
9	40-year-old male	The patient was admitted with abdominal pain and jaundice found to have BL with lymphomatous infiltration of the gallbladder and common bile duct.	[14]
10	21-year-old male	The patient presented with abdominal pain, abdominal distention, weight loss, and jaundice. The patient was diagnosed with primary pancreatic BL.	[15]

## Conclusions

In conclusion, primary pancreatic BL is a rare and aggressive neoplasm that should be considered in the differential diagnosis of young patients presenting with obstructive jaundice. Early recognition is pivotal as it facilitates the timely implementation of tailored treatment approaches, which in turn leads to enhanced clinical outcomes and an improved overall prognosis for affected individuals. Medical professionals must maintain a high index of suspicion, utilize advanced diagnostic modalities, and collaborate across disciplines to ensure optimal care for patients with primary pancreatic BL.
